# Changes in Seasonal Patterns of Pediatric Respiratory Viral Infections Before, During, and After the COVID-19 Pandemic: A Seventeen-Year Surveillance Study in the Republic of Korea

**DOI:** 10.3390/v18040420

**Published:** 2026-03-29

**Authors:** Mi-Ru Oh, Jeong Su Han, Jae-Sik Jeon, Jae Kyung Kim

**Affiliations:** Department of Biomedical Laboratory Science, College of Health Sciences, Dankook University, Cheonan-si 31116, Republic of Korea; mrmr1028@naver.com (M.-R.O.); jshan1162@naver.com (J.S.H.); zenty87@naver.com (J.-S.J.)

**Keywords:** pediatric respiratory viruses, COVID-19, multiplex polymerase chain reaction, human rhinovirus, human parainfluenza virus type-3, respiratory syncytial virus

## Abstract

The coronavirus disease 19 pandemic disrupted pediatric respiratory infections through non-pharmaceutical interventions and altered contact patterns. Long-term comparisons across the pandemic timeline in children remain limited. In this study, we analyzed 15,657 respiratory specimens from patients ≤ 18 years at Dankook University Hospital (2007–2023) using multiplex polymerase chain reaction assays targeting 15 viruses. Age-stratified positivity rates were compared across pandemic phases. Children ≤ 6 years comprised 88.61% of the study population. Human rhinovirus showed the highest detection rate (24.06%), followed by adenovirus (12.33%), respiratory syncytial virus-subtypes A and B (RSV-A: 11.13%; RSV-B: 8.65%), human parainfluenza virus-type 3 (HPIV-3; 6.21%), human metapneumovirus (HMPV; 5.33%), and enterovirus (2018–2023; EV; 10.96%). Monthly distributions differed (*p* < 0.001). RSV peaked in late autumn and winter; influenza and seasonal coronaviruses in winter and spring; HMPV, HPIV-3, EV, and human bocavirus in summer and fall. Positivity declined during the pandemic, rebounding in 2023, most prominently among children aged 1–6 years (84.91%). HPIV-3 and EV increased (*p* < 0.001). RSV-A predominated pre-pandemic, whereas RSV-B showed a non-significant relative increase post-pandemic; no subtype differences occurred during the pandemic. Findings demonstrate pathogen-specific shifts in predominance and seasonality and support ongoing surveillance and pediatric care planning.

## 1. Introduction

A constant influx of neonates with underdeveloped immunity makes the overall pediatric population particularly vulnerable to infections. Furthermore, respiratory viruses are the primary cause of acute respiratory infections in this population [1]. In infants and young children, progression to lower respiratory infections, such as bronchiolitis and pneumonia, is common. These illnesses place substantial demands on acute care, including emergency visits, hospitalizations, and respiratory support [2].

Human parainfluenza virus (HPIV) is a major cause of laryngotracheobronchitis and can lead to lower respiratory tract infections [3]. Respiratory syncytial virus (RSV) is a key pathogen responsible for severe lower respiratory tract infections and seasonal epidemics in infants and young children [4]. Other viruses, including influenza, human rhinovirus (HRV), adenovirus (Adeno), human bocavirus (HBoV), and human coronaviruses (HCoVs), such as OC 43, Cov 229E, and NL63, show consistent seasonal patterns in children. These viruses form dominant structures within the pediatric respiratory virus ecosystem, shaped by age-specific sensitivity, transmission characteristics, immune persistence, and environmental factors [5].

However, the coronavirus disease 2019 (COVID-19) pandemic, which began in late 2019, has fundamentally altered seasonal respiratory virus patterns [6]. Non-pharmaceutical interventions (NPIs), including mask use, distancing, and school or attendance restrictions, have drastically reduced the circulation of multiple endemic respiratory viruses while simultaneously reshaping contact patterns and trajectories of immune accumulation [7].

Resurgence during the mitigation phase represents more than a return to prior patterns. Epidemic periods shifted, epidemic timing/patterns were reorganized, relative dominance among pathogens changed, and the burden refocused childhood age cohorts [8]. This phenomenon, typically termed an “immunity gap,” occurs when population susceptibility accumulates during a pandemic. The subsequent restoration of social contacts can subsequently result in atypical epidemic sizes [9]. Children are particularly affected because immunity develops rapidly with age, and severe infections have a clinically significant impact.

In the Republic of Korea, high levels of NPIs were implemented during the pandemic. Concurrently, declining birth rates and rapid changes in the childhood population structure highlight the importance of monitoring the pediatric respiratory viral burden. This situation requires careful monitoring and medical resource allocation to evaluate how the pediatric respiratory viral burden has been reconstructed across pre-pandemic, pandemic, and post-pandemic recovery periods [10,11].

Tracking monthly epidemics of multiple pathogens over long periods requires a consistent observation framework. This framework must account for time-series confounders, such as changes in test volume, testing indications, healthcare utilization, and test panels.

Research has indicated that the relative dominance of RSV subtypes A and B (RSV-A and RSV-B) or HPIV-3 genotypes may shift, along with changes in the epidemic timing. Therefore, assessing viral dominance cannot rely solely on variations in positivity rates [12,13]. Against this backdrop, long-term single-center surveillance time series provide a structured framework to detect how pandemic-related disruptions emerge within a consistent observation system. Such data elucidate when epidemics occur, which pathogens predominate, and which age cohorts are most affected.

Multiplex polymerase chain reaction (PCR) surveillance enables simultaneous detection of multiple pathogens within the same clinical setting. This approach supports comprehensive assessment of individual viral changes, relative pathogen composition, and age-specific shifts. Therefore, understanding post-pandemic pediatric respiratory viral epidemiology requires quantifying epidemic timing, pathogenic dominance, and age-related burden, rather than focusing solely on whether epidemics rebound.

In this study, we used surveillance data from respiratory specimens collected between 2007 and 2023 at a single-center tertiary medical institution in the Republic of Korea. We aimed to evaluate changes in pediatric respiratory viral epidemiology across the pre-pandemic, pandemic, and post-pandemic recovery periods among individuals aged ≤18 years old. In particular, we quantified overall and virus-specific monthly positivity rates over a 17-year time series and examined changes in epidemic timing.

Moreover, we compared the relative dominance of major viruses, including HPIV, RSV, influenza virus (Inf), enterovirus (EV), HRV, Adeno, HBoV, and seasonal HCoVs. Age-specific redistribution of disease burden was evaluated, and changes in the relative proportion and predominance of RSV-A and RSV-B were analyzed across periods. We designed these objectives to strengthen pediatric respiratory infection surveillance, refine testing strategies, and guide post-pandemic medical resource allocations.

## 2. Materials and Methods

### 2.1. Ethical Considerations

This study was conducted in accordance with the ethical principles of the Declaration of Helsinki of 1975, which was revised in 2013, and STROBE reporting guidelines (Table S1). Furthermore, this study was approved by the Institutional Bioethics Committee of Dankook University on 23 April 2025, under the protocol approval number: DKU 2025-02-004-003. Because this retrospective analysis used de-identified data, the requirement for informed consent was waived.

### 2.2. Research Design

In this single-center retrospective observational study, we evaluated the long-term changes in 15,657 respiratory virus test results from pediatric patients aged ≤18 years old. These patients underwent multiplex PCR testing at Dankook University Hospital between January 2007 and December 2023 when they presented with respiratory symptoms, and an acute viral respiratory infection was clinically suspected by the treating physician.

Data were extracted from the Laboratory Information System of the Department of Diagnostic Laboratory Medicine and linked to age and test date (month and year) at the time of testing through electronic medical records (EMRs).

### 2.3. Variable Definitions and Seasonal Classifications

In this study, age was calculated based on the test date and used as an analysis variable. As per the Korea Centers for Disease Control and Prevention criteria for classifying children’s ages, children were divided into four cohorts: <1 year old, 1–6 years old, 7–12 years old, and 13–18 years old [14].

Time variables included the year, month, and pandemic period. Months were assigned based on test dates, and monthly positivity rates and epidemic windows were evaluated. The peak month was defined as the month with the highest positivity. Seasons were classified into spring (March–May), summer (June–August), fall (September–November), and winter (December–February). The pandemic timeline was divided into pre-pandemic (2007–2019), pandemic (2020–2022), and post-pandemic recovery (2023) periods, to assess the impact of the COVID-19 pandemic on respiratory virus circulation.

### 2.4. Laboratory Testing

Respiratory virus detection was performed using a multiplex PCR panel targeting 15 viruses. Nasopharyngeal specimens were collected using sterile cotton swabs, inspected according to the internal laboratory standard operating procedures, and stored at 4 °C for ≤24 h before analysis. Viral nucleic acids were extracted using a QIAamp Viral RNA Mini Kit (QIAGEN, Hilden, Germany) according to the manufacturer’s instructions, and the multiplex panel included targets for RNA and DNA respiratory viruses. All multiplex PCR assays were performed in the hospital diagnostic laboratory as part of routine clinical testing under established internal quality-control procedures and external quality-assurance practices.

Between 2007 and 2012, respiratory viruses were detected using the Seeplex™ RV series multiplex PCR assay (Seegene, Seoul, Republic of Korea). Results were confirmed by gel electrophoresis after conventional endpoint PCR. Sequencing was not performed during this period to confirm the PCR products. After 2013, multiplex real-time reverse transcription (RT)-PCR tests were conducted, as per the manufacturer’s instructions. The tests used the AdvanSure™ RV and RV-Plus Real-Time RT-PCR kits (LG Life Sciences, Seoul, Republic of Korea) with the SLAN Real-Time PCR System (LG Life Sciences, Seoul, Republic of Korea). Previous evaluation studies have reported broadly comparable overall performance between Seeplex respiratory virus assays and the AdvanSure real-time RT-PCR assay [15,16].

Given the shift in testing methodology, the study focused on relative comparisons of epidemic timing, window, and duration. According to panel composition, EV results were available from 2018 to 2023, and NL63 and HBoV results from 2015 to 2023.

### 2.5. Data Analysis

Patient age was summarized using the median and interquartile range (IQR; 25–75th percentiles), because the age distribution was concentrated in younger children and was not assumed to be normally distributed. The IQR (Q3–Q1) was used as a measure of variability around the median.

Monthly seasonality was evaluated for each virus using Pearson’s chi-square test for independence with 12-month categories (degrees of freedom [df] = 11), based on the number of positive cases and total tests per month. To quantify seasonality beyond the presence of a monthly effect, a binary logistic regression model, including first- and second-harmonic terms, was fitted, treating the month as a cyclic variable. Model selection was performed based on the Akaike Information Criterion (AIC). Comparisons between overlapping models, such as the inclusion of harmonic terms, were evaluated using the likelihood ratio test.

Pearson’s chi-square test was used to compare age cohorts and pandemic periods (pre-pandemic, pandemic, and post-pandemic recovery). For virus-specific comparisons across the three periods, omnibus *p* values were derived from Pearson’s chi-square tests of independence; Fisher’s exact test was used when expected cell counts were <5. To account for multiple virus-specific comparisons across the three study periods, *p* values were adjusted using the Benjamini–Hochberg false discovery rate procedure. Differences between periods were summarized as odds ratios (ORs) with 95% confidence intervals (95% CIs). To complement the omnibus comparisons across the three study periods, pairwise comparisons were additionally performed for pre-pandemic vs. pandemic, pre-pandemic vs. post-pandemic, and pandemic vs. post-pandemic using Fisher’s exact test. ORs with 95% CIs and Holm-adjusted *p* values were calculated for these comparisons.

Regarding the RSV-subtype analysis, a binary logistic regression including a period × subtype interaction was fitted. The OR and 95% CI of RSV-B relative to RSV-A were calculated for each period. Furthermore, 95% CIs for the ratios were computed using the Wilson method.

To minimize biases from denominator fluctuations arising from changes in test methodologies or panel compositions over time, the analysis range for each virus was restricted based on coverage. Viruses with limited coverage, such as HBoV, EV, and NL63, were analyzed and interpreted only within the period after their introduction.

Comparisons across pandemic periods (pre-pandemic, pandemic, post-pandemic) were conducted using a complete-case–cohort. This cohort included only samples for which common panel results and key variables, such as patient age and test date, were consistently available across all three periods. Consequently, during the panel transition in 2023, only samples meeting these criteria were included, and the denominator was relatively reduced. Period-comparative analyses were conducted using the final cleaned analytical dataset, which included only samples with complete information on the key variables.

Each specimen case was treated as a separate unit of analysis. Repeated tests from the same patient were counted as separate cases. All statistical analyses were performed using the R software (R Foundation for Statistical Computing, version 4.3.3, Vienna, Austria). Statistical significance was set at *p* < 0.05.

## 3. Results

### 3.1. Patient Characteristics and Overall Positivity Rate

**Figure 1:**
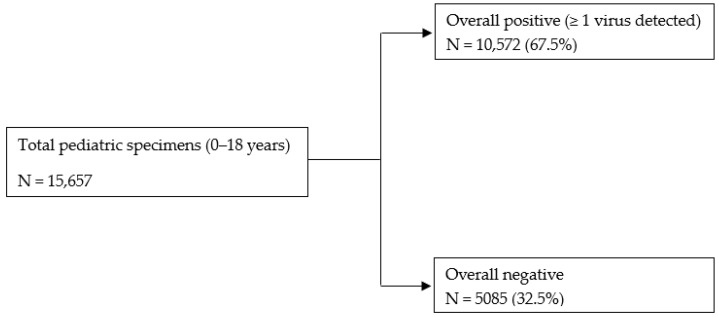
Study Flow of Specimens From Pediatric specimens by Overall Viral Detection Status From 2007 to 2023.

Data were collected from a total of 15,657 specimens at Dankook University Hospital between 2007 and 2023. The composition of the study population and classification flow of virus detection results (positive or negative) are presented in Figure 1. The age distribution was primarily concentrated in infants and toddlers, with 4556 (29.10%) and 9317 (59.51%) specimens in the <1- and 1–6-year-old cohorts, respectively. Thus, 88.61% of the total study population were <6 years old.

The remaining age cohorts included 1207 specimens (7.71%) aged 7–12 years old and 577 specimens (3.69%) aged 13–18 years old. The median age was 1.0 years old, with an IQR of 0.37–3.0 years old (Table 1). Sex distribution included 9032 male (57.69%) and 6625 female (42.31%) specimens.

### 3.2. Virus-Specific Detection Rates and Coverage Analyses

**Figure 2:**
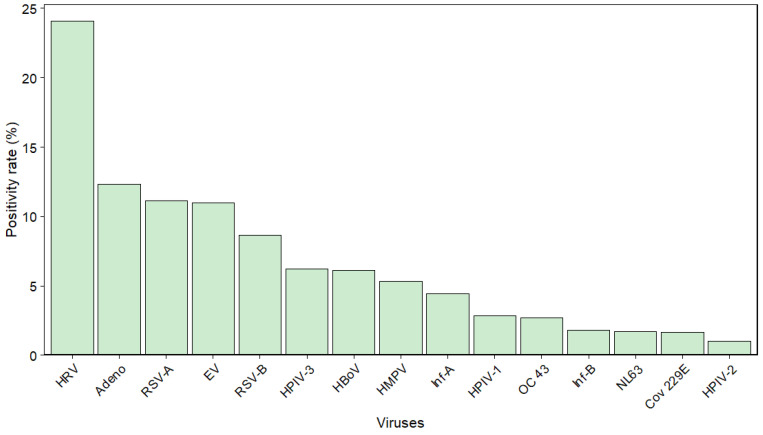
Virus-Specific Positivity Rates of Pediatric Specimens (2007–2023), Adjusted for Panel Coverage. Bars represent the overall virus-specific positivity rates (%) of the specimens from pediatric patients ≤ 18 years old. Abbreviations: Inf = influenza virus; RSV = respiratory syncytial virus; HMPV = human metapneumovirus; HPIV = human parainfluenza virus; HRV = human rhinovirus; HCoV = human coronavirus; Adeno = adenovirus; HBoV = human bocavirus; EV = enterovirus. HCoV includes the subtypes OC 43, 229E, and NL63.

Analysis of 15,657 specimens showed the highest detection rates for HRV at 24.06% (95% CI: 23.40–24.74), followed by Adeno (12.33%; 95% CI: 11.82–12.85), RSV-A (11.13%; 95% CI: 10.64–11.63), RSV-B (8.65%; 95% CI: 8.22–9.10), HPIV-3 (6.21%; 95% CI: 5.84–6.60), and human metapneumovirus (HMPV; 5.33%; 95% CI: 4.99–5.69) (Figure 2).

Detection rates for NL63 and HBoV were calculated for 2015–2023 (n = 6552), and for EV for 2018–2023 (n = 2355), yielding 1.71% (95% CI: 1.42–2.05), 6.14% (95% CI: 5.58–6.74), and 10.96% (95% CI: 9.76–12.28), respectively (Table 2).

### 3.3. Monthly and Seasonal Trends

**Figure 3:**
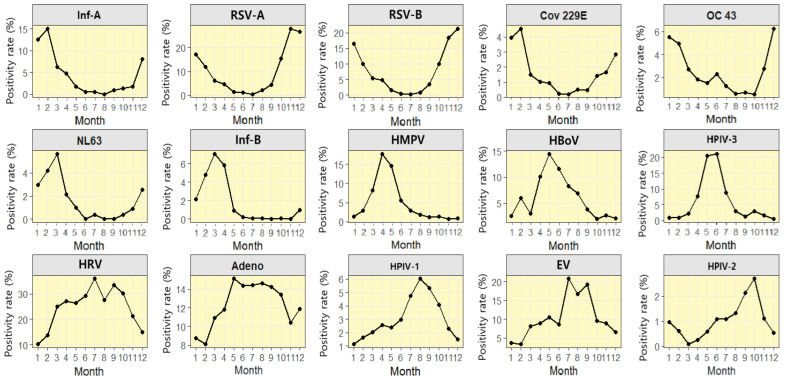
Monthly Positivity Profiles of 15 Respiratory Viruses Across the Calendar Year. Abbreviations: Inf = influenza virus; RSV = respiratory syncytial virus; HMPV = human metapneumovirus; HPIV = human parainfluenza virus; HRV = human rhinovirus; HCoV = human coronavirus; Adeno = adenovirus; HBoV = human bocavirus; EV = enterovirus. HCoV includes the subtypes OC 43, 229E, and NL63. Each panel shows the observed monthly positivity rate (%) for each virus. The x-axis indicates the month (1–12), and the y-axis shows the positivity rate (%) on virus-specific scales. This approach captures within-year (12-month) seasonal patterns, complementing single-year post-pandemic recovery period data. Monthly positivity rates for EV have been calculated using data from 2018 to 2023. Monthly positivity rates for NL63 and HBoV have been calculated using data from 2015 to 2023, reflecting differences in assay panel coverage over time.

The monthly positivity rate distribution differed statistically significantly across months (January–December) and among the 15 viral strains analyzed for detection (*p* < 0.001; Figure 3; Table 3).

When seasonal dominance patterns were summarized using monthly positivity rates, RSV-A and RSV-B showed distinct winter peaks. RSV-A peaked in November (27.85%) and remained high in December (26.47%), with a seasonal window spanning late fall to early winter. RSV-B peaked in December (21.30%) and remained elevated in November (18.35%) and January (16.49%), reflecting late fall to mid-winter dominance. Inf-A peaked in February (15.08%) and remained concentrated throughout January and February. Among seasonal HCoVs, Cov 229E peaked in February (4.55%), indicating winter dominance, whereas OC 43 peaked in December (6.24%) and January (5.50%), with this dominance centered in winter.

NL63 had a single peak in March (5.65%), with continued detection during the period of January–February (2.95–4.19%) and December (2.52%), indicating a distribution from late winter to early spring. Inf-B peaked in March (7.07%) and remained elevated in April (5.84%), confirming dominance in early spring. HMPV circulated mainly in spring (April–May), peaking in April (17.78%) and remaining high in May (14.62%). Dominance extended from late spring to early summer. HBoV peaked in May (14.49%) and remained elevated for the period of April–June, indicating late spring to early summer dominance. HPIVs showed subtype differences. HPIV-3 was concentrated in late spring to early summer, peaking in May (20.50%), whereas HPIV-1 peaked in August (6.03%).

HRV was detected throughout the year, with a clear peak in July (35.98%) and predominance from June to August. Elevated levels persisted through September and October. Additionally, Adeno circulated throughout the year yet remained relatively high from May to October (13.44–15.14%), consistent with summer to early fall predominance.

EV peaked in July (20.83%) and remained elevated in August and September, confirming summer to early fall (July–September) predominance. HPIV-2 had a low overall positivity rate, with its peak occurring in October (2.70%), indicating a relatively dominant pattern in fall (September–November).

In this section, peak months refer to observed monthly maxima based on empirical monthly positivity rates. In contrast, Table S2 reports model-estimated peak months derived from harmonic logistic regression.

Peak months and seasonal intensities were calculated to quantitatively supplement the monthly observation patterns (Table S2). Inf-B showed the strongest seasonality. RSV and inf-A peaked in late fall to winter, whereas NL63 showed peaks in winter to early spring.

HMPV, HPIV-3, and HBoV peaked in spring to early summer, whereas HRV and Adeno showed relatively weak seasonality (*p* < 0.001). Because the fitted harmonic curve summarizes the overall seasonal pattern, the model-estimated peak month may differ from the observed monthly maximum in viruses with broad seasonal plateaus, asymmetric seasonal shapes, or similar positivity across adjacent months.

### 3.4. Trends in Positivity Rates by Age Cohorts and Study Periods

Positivity rates decreased across all age cohorts during the pandemic and showed an overall increase in the post-pandemic recovery period. Among children aged <1 year old, rates declined from 64.55% to 44.86% before rising to 57.50%. For those aged 1–6 years old, rates decreased from 74.40% to 68.72% before increasing to 84.91%. In the 7–12-year-old cohort, rates fell from 45.79% to 31.25%. Adolescents aged 13–18 years old showed a decline from 37.38% to 17.02%, followed by an increase to 45.45%. Differences in the positivity rates across periods (pre-pandemic, pandemic, post-pandemic recovery) were statistically significant across all age cohorts (<1 year old: *p* < 0.001; 1–6 years old: *p* = 0.00231; 7–12 years old: *p* = 0.0267; 13–18 years old: *p* = 0.011). Within each period, positivity rates notably differed among age cohorts (all periods: *p* < 0.001) (Table 4). Additional pairwise comparisons across the three period contrasts within each age cohort are provided in Table S3.

### 3.5. Dominance of HPIV-3: Major Post-Pandemic Changes

**Figure 4:**
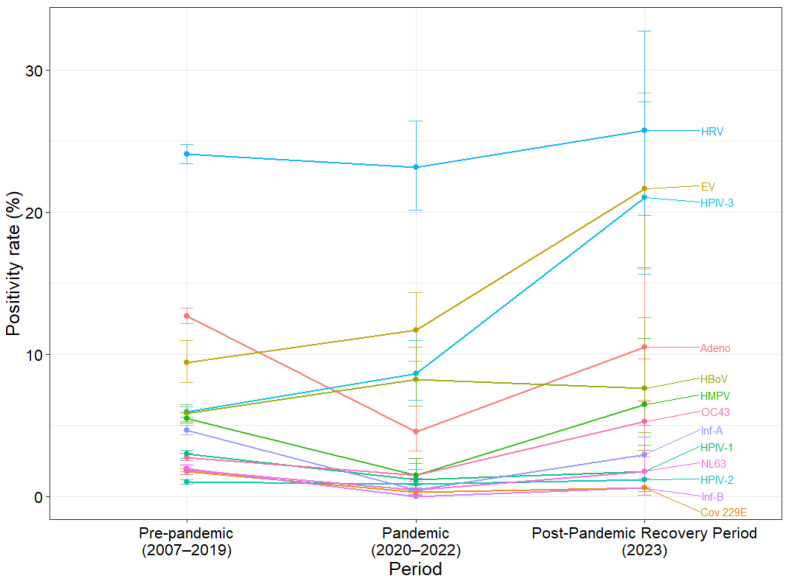
Virus-specific positivity rates (%) across three periods with 95% confidence intervals: pre-pandemic (2007–2019), pandemic (2020–2022), and post-pandemic recovery (2023). Points represent positivity rates, and error bars denote 95% confidence intervals.

Among the 13 non-RSV viruses, positivity rates changed considerably across the pre-pandemic (2007–2019), pandemic (2020–2022), and post-pandemic recovery (2023) periods for 11 viruses, whereas HRV and HPIV-2 showed no notable change (Table 5). The RSV-A and RSV-B subtypes are described separately in Section 3.6. HPIV-3 showed the largest increase, rising from 5.92% (87/14,803) pre-pandemic to 21.05% (36/171) post-pandemic (Figure 4). The odds of HPIV-3 positivity were statistically significantly higher in the post-pandemic period compared with the pre-pandemic period (OR: 4.23, 95% CI: 2.91–6.15; *p* < 0.001).

EV positivity increased from 9.39% (141/1501) in the pre-pandemic period to 21.64% (37/171) in the post-pandemic period (omnibus *p* < 0.001), with a notable OR of 2.66 (95% CI: 1.78–3.99; *p* < 0.001). OC 43 rose from 2.75% (407/14,803) pre-pandemic to 5.26% (40/171) post-pandemic; however, this increase was not statistically significant (OR: 1.97, 95% CI: 1.00–3.87; *p* = 0.057).

After adjustment for multiple virus-specific comparisons using the Benjamini–Hochberg false discovery rate procedure, statistically significant differences across periods remained for all viruses except HRV and HPIV-2. However, in the direct comparison between the post-pandemic recovery period and the pre-pandemic period, only HPIV-3 and EV remained significantly increased after adjustment (Table S4).

Additional pairwise comparisons across the three period contrasts for each virus are provided in Table S5.

### 3.6. Relative Contribution of RSV Subtypes, Particularly RSV-B

**Figure 5:**
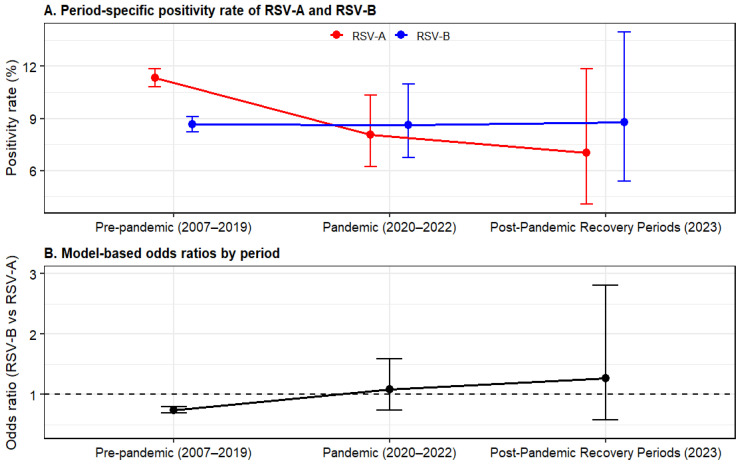
Period-Specific Shifts in Respiratory Syncytial Virus (RSV)-A and RSV-B Positivity and Model-Based Subtype Odds Ratios (ORs) Across the Pre-Pandemic, Pandemic, and Post-Pandemic Recovery Periods. (**A**) Positivity rates (%) of RSV-A (red) and RSV-B (blue) by period, with 95% Wilson confidence intervals (CIs). (**B**) Model-based ORs comparing RSV-B with RSV-A by period. Points represent ORs, and whiskers indicate 95% CI. The dashed line denotes OR = 1, indicating no difference between subtypes. OR > 1 indicates higher odds for RSV-B. OR < 1 indicates higher odds for RSV-A.

Focusing on RSV-A and RSV-B, we evaluated changes in subtype predominance and the proportion of RSV-B over time. Positivity rates and relative predominance of RSV-A and RSV-B varied across periods. Pre-pandemic (2007–2019), RSV-A positivity was 11.32% (1675/14,803; 95% CI: 10.81–11.84), higher than RSV-B positivity at 8.65% (1281/14,803; 95% CI: 8.21–9.12). During the pandemic (2020–2022), RSV-A and RSV-B positivity were similar at 8.05% (55/683) and 8.64% (59/683; 95% CI: 6.76–10.98), respectively. In the post-pandemic recovery period (2023), RSV-B positivity (8.77%, 15/171; 95% CI: 5.39–13.97) was numerically higher than RSV-A positivity (7.02%, 12/171; 95% CI: 4.06–11.86) (Figure 5).

The OR comparing RSV-B with RSV-A was 0.742 (95% CI: 0.688–0.802; *p* < 0.001) pre-pandemic, indicating statistically significantly lower odds of RSV-B positivity relative to RSV-A positivity. Conversely, during the pandemic, no distinction between subtypes was observed (OR: 1.080; 95% CI: 0.736–1.585; *p* = 0.696). During the post-pandemic recovery period, the OR was 1.274 (95% CI: 0.578–2.809; *p* = 0.548), suggesting higher odds of RSV-B positivity; nonetheless, the difference was not statistically significant (Table 6).

## 4. Discussion

In this study, we analyzed 17 years of pediatric respiratory virus PCR surveillance data from a single center in Korea to characterize ecological shifts across the pre-pandemic, pandemic, and post-pandemic recovery periods. Surveillance studies from Canada and China have reported marked declines in major respiratory virus circulation during the COVID-19 pandemic, followed by post-pandemic resurgence and temporal shifts [17,18]. Similar patterns have been documented in Korea, with proposed explanations, including an immunity gap, restoration of contact patterns, and rebalancing of viral competition [19].

In addition to these epidemiologic mechanisms, the observed post-pandemic increase in viral detections should also be interpreted in the context of healthcare utilization and testing practice. During the pandemic, healthcare-seeking behavior for pediatric respiratory symptoms may have been reduced because of public concern, restricted access, and changes in thresholds for hospital visits. As routine medical use resumed in the recovery period, children with respiratory symptoms may have been more likely to present for evaluation, and clinicians may have resumed broader multiplex PCR testing. Accordingly, the post-pandemic increase in viral detections may reflect not only true epidemiologic rebound but also the normalization of healthcare-seeking behavior, testing volume, and clinical testing indications.

The present long-term dataset in this study confirms these patterns in the pediatric population and further demonstrates that post-pandemic recovery trajectories differ by pathogen.

Infants and toddlers comprised a large proportion of the study population, which informs the interpretation of the findings. Neonates receive maternal antibodies against respiratory viral infections through transplacental transfer, providing temporary protection. This passive immunity declines rapidly during early life. The resulting reduction in protection likely alters age-specific susceptibility patterns [20,21].

Virus-specific positivity rates indicate that pediatric respiratory infections are not driven by a single pathogen. A stratified spectrum emerges, with HRV and Adeno forming the baseline burden, whereas seasonal viruses increase disease burden during defined periods. HRV occupies the highest position within this structure, reflecting sustained transmissions in households and community settings.

Previous studies in Korea and the United States of America (USA) have noted that some pathogens maintain circulation with limited variation, particularly during exogenous shocks, such as the pandemic [22,23]. By contrast, seasonal viruses have exhibited distinct peaks and large fluctuations in epidemic intensity, corresponding with changes in contact patterns and gaps in population immunity. Therefore, the transitional rearrangement observed in this study does not represent a full return to pre-pandemic epidemic patterns. Selective enhancement and restructuring occurred in the seasonal layer above the year-round baseline burden.

The viral spectrum directly influences clinical surveillance operations. Year-round baseline viruses drive consistent diagnostic demand and contribute to the burden in patients with respiratory symptoms. Seasonal viruses generate short-term surges in testing, hospital beds, and isolation resources during their epidemic windows [24,25]. Post-pandemic, shifts in the timing of these epidemic windows require pathogen-specific prioritization of testing and resource allocation rather than relying solely on general ranking information.

Monthly epidemic patterns shift the focus from a “single peak” to a defined “epidemic window.” In this study, RSV activity was concentrated in the late fall to winter period, consistent with reported findings from the USA Centers for Disease Control and Prevention [26]. Influenza and seasonal HCoVs are known to peak in winter to early spring [27]. Moreover, HMPV and HPIV-3 exhibit activity in spring to early summer, whereas EVs show increased activity in summer to early fall [28,29]. Epidemic windows have practical implications, directly indicating periods of heightened testing demand, hospital bed use, and isolation operations for policy and clinical planning.

Comparison of the periods by age cohort showed a common trajectory across all cohorts, with a decrease during the pandemic and a rebound in the post-pandemic recovery period. In children, immune experiences accumulate rapidly with age, and reduced exposure during the pandemic may have delayed superinfections and expanded the susceptible population [30]. This accumulation of susceptibility, combined with restored opportunities for transitional contact, promoted epidemic resumption and, for some pathogens, apparent reinforcement of activity. Infections are redistributed into specific age cohorts and environments with dense contact, such as childcare facilities and schools, which function as major nodes for childhood transmission [31]. Reduced exposure during the pandemic likely affected the accumulation of immune experiences in infancy and early infection dynamics, explaining the shift in burden toward younger cohorts during the recovery period. The simultaneous increase across all age cohorts during this recovery period suggests that susceptibility and exposure opportunities in the pediatric population were reorganized collectively.

However, the relatively higher positivity observed in the 7–12 and 13–18 age groups during the post-pandemic period should be interpreted cautiously. Because pairwise comparisons between the pre-pandemic and post-pandemic periods were not statistically significant in these age groups, these findings are better regarded as descriptive patterns rather than definitive evidence of an age-specific post-pandemic increase. They may reflect not only epidemiologic changes but also reduced testing volume, selective testing of more symptomatic patients, and changes in healthcare-seeking behavior and test utilization after the pandemic.

Among the transitional pathogens, HPIV-3 showed the most pronounced structural change. Although the immunity gap may explain the rebound of several pathogens, the relative prominence of specific viruses is likely to reflect the combined effects of transmission efficiency, immune persistence, age-specific contact patterns, and changes in exposure opportunities before and after the pandemic. These findings suggest that the post-pandemic increase in HPIV-3 was at least partly associated with a reorganization of epidemic timing. At the same time, the magnitude of the increase may also reflect a rebound in transmission intensity as contact patterns normalize. However, this finding should not be interpreted as resulting from a single mechanism, because changes in healthcare utilization during the recovery period, seasonal testing volume, and clinical indications for testing may also have contributed to the increase in HPIV-3 positivity. Similar changes in epidemic patterns have been reported in other surveillance studies, supporting the possibility of true epidemiologic reorganization [32]. Nevertheless, because this study was based on retrospective single-center data, it was difficult to completely disentangle the relative contribution of testing-related factors. Therefore, further multi-center studies integrating testing utilization, clinical severity, and molecular epidemiologic data are needed to more clearly elucidate the drivers of post-pandemic HPIV-3 resurgence. The increase in EVs may also be interpreted in a similar context.

Regarding RSV, subtype predominance fluctuated across the pre-pandemic, pandemic, and post-pandemic recovery periods. Pre-pandemic, RSV-A predominated, yet during the pandemic, the gap between RSV-A and RSV-B narrowed. In the post-pandemic recovery period, RSV-B showed a relative increase. However, because statistical significance was not reached during this period, this change should be interpreted as a directional signal rather than a decisive shift.

Moreover, surveillance studies in Bulgaria and Austria have documented post-pandemic RSV-B predominance, indicating that immune gaps and changes in contact patterns may leave lasting effects on RSV epidemiology [33,34]. The relative shifts between subtypes observed in this study likely reflect a combination of factors. First, the subtype-specific immune landscape may have flattened following reduced exposure. Second, the predominance of initial introductions could have caused fluctuations at the onset of the epidemic window. Third, potential differences in propagation fitness at the subtype or genotype level may have existed. Therefore, subtype-level monitoring can detect compositional changes that a single positive rate may miss and can inform targeted prevention strategies, including the timing of interventions and allocation of resources.

Given that RSV activity remained concentrated in the late fall to winter period, RSV vaccination and other preventive interventions may benefit from being scheduled before the onset of the epidemic window rather than being tied to a fixed calendar alone. In this context, early autumn may represent a reasonable time for consideration, although the optimal timing should ideally be guided by local surveillance data.

The clinical and policy implications can be summarized as adaptive surveillance. During the post-pandemic recovery period, the epidemic window was not fixed. Some years saw an earlier epidemic onset or shifted peak. The surveillance system should be able to flexibly adjust test frequency, target population, and resource allocation to reflect changing infection patterns.

In hospital-based surveillance, this approach can be operationalized by regularly monitoring monthly virus-specific positivity, testing volume, and age-stratified detection patterns. Early deviation from the expected seasonal baseline may serve as an operational trigger for timely adjustment of testing strategies, laboratory surge capacity, staffing and reagent allocation, and isolation resources according to the pathogens and age groups most affected. In this way, hospitals may respond more rapidly and flexibly to shifts in epidemic timing and intensity than would be possible under fixed calendar-based assumptions.

When early rising signals are detected, preemptive reallocations of testing capacities and hospital isolation resources for pediatric outpatients and emergencies are necessary. This approach enables early detection and intervention while minimizing resource waste or shortages. If signs of increased activity for HPIV-3 and HMPV appear in spring and early summer, testing priorities, laboratory turnaround targets, isolation bed plans, and staffing should be aligned with the season. This prevents the concentration of testing and hospital demand solely in winter.

By contrast, RSV, influenza, and seasonal HCoVs have relatively clear winter to early spring windows. Early testing and isolation strategies should be advanced when the epidemic begins earlier in the year. EVs, which peak in summer to early fall, should be monitored in coordination with school and childcare schedules. Diagnostic capacity and surveillance intensity should be adjusted to account for possible increases in emergency visits and mass outbreaks.

This epidemic window-based operation supports the prediction of surge timing and helps minimize simultaneous resource shortages, rather than relying on annual summaries. In addition, RSV prevention strategies benefit from optimizing timing and target age according to shifts in epidemic timing and intensity. The long-term data in this study provide a regional perspective on modified patterns pre- and post-pandemic, informing the development of tailored strategies.

However, this study had some limitations. The analysis reflects a single-center retrospective observational design, limiting regional generalizability. As a single-center hospital-based study, this analysis may also be subject to selection bias. The study population consisted of children who presented for medical evaluation and underwent multiplex PCR testing and therefore may overrepresent specific age groups, clinical severities, or referral patterns. In addition, hospital-based surveillance does not necessarily reflect community-level viral circulation, because healthcare-seeking behavior, access to care, symptom severity, and testing indications influence which patients are tested. Accordingly, the observed viral detection patterns should be interpreted as reflecting trends within a hospital-based pediatric testing population rather than the full epidemiology of respiratory virus circulation in the community.

Long-term trends may have been influenced not only by underlying epidemiologic changes but also by changes in healthcare-seeking behavior, test-referral criteria, testing volume, and clinical indications for multiplex PCR across the pre-pandemic, pandemic, and recovery periods. Temporal bias or misclassification may have arisen from variations in test indications, sample transport conditions, PCR kit versions or protocols, and reading algorithms, over the 17-year observation period.

Testing methodologies changed from endpoint PCR (2007–2012) to real-time RT-PCR (after 2013), which could have affected absolute positivity rates as a result of differences in detection sensitivity and panel composition. Although previous evaluation studies have reported broadly comparable overall performance between the Seeplex and AdvanSure respiratory virus assays, differences in assay chemistry, analytical sensitivity, and panel composition may still have influenced absolute positivity estimates across years. The 2020–2022 interval was defined as the pandemic period; however, international intensity and timing of NPIs and medical use patterns likely varied within that period.

Limited clinical information, including disease severity, hospitalization status, and detailed testing indications, limited our ability to distinguish true increases in viral circulation from changes in patient selection and testing practices. High positivity rates may occur in patients with severe conditions, whereas low rates may appear in patients with mild conditions or outpatients. Environmental, behavioral, and socioeconomic factors, such as temperature, humidity, population density, and healthcare access, could not be directly assessed, limiting adjustment for potential confounders.

The post-pandemic recovery year (2023) provided an initial signal of epidemic rebound and movement rather than a stable post-pandemic equilibrium. Estimates based on the recovery period may have overestimated or underestimated the magnitude of changes because of increased uncertainty.

Nevertheless, in this study, we emphasized relative rearrangements during the pandemic. We focused on changes in the pandemic window, relative predominance pattern, and age-specific burden pre- and post-pandemic. However, our analysis did not prioritize absolute values. Coverage-based denominator processing (Table 2) and legacy panel intersection analysis (Table 4) minimized biases and ensured comparability of transition data.

Future research should include multiple institutions or national units to verify whether epidemic windows and timing rearrangements can be reproduced in the same temperate Asian environment. Studies are needed to determine whether the increase in HPIV-3 results from specific genotype introduction and dominance, antigenic variation, or changes in transmission networks using molecular epidemiological data. RSV testing can help clarify the dynamics of subtype intersections by combining subtype and genotype monitoring with estimation of introduction points.

Analyses that include severity indicators, such as hospitalization, respiratory support, and intensive care treatment through EMR linkages, are essential to assess whether trend rearrangements affect medical burdens. Furthermore, modeling incorporating environmental and behavior variables is required. Using generalized additive or variance delay models can quantify the contributors to epidemic window shifts. Key factors to include are temperature, school schedules, and population density, which can influence access to medical care. Monthly positivity rates serve as dependent variables in these models.

Overall, the results indicate that post-pandemic pediatric respiratory virus activity did not simply return to the pre-pandemic pattern. Instead, period-specific differences were observed in circulation patterns across viruses and age groups. In particular, HPIV-3 and RSV subtypes showed distributions that differed from those seen before the pandemic. These findings may be consistent with changes in population immunity and contact patterns, although the present study was not designed to determine the underlying mechanisms directly. Accordingly, monitoring and prevention strategies may need to remain responsive to real-time epidemiologic signals rather than relying solely on fixed seasonal expectations.

## 5. Conclusions

Based on long-term pediatric respiratory virus surveillance at a single institution from 2007 to 2023, the findings of this study suggest that post-pandemic pediatric respiratory virus activity did not simply return to the pre-pandemic pattern. Instead, period-specific differences were observed in circulation patterns across viruses and age groups. In particular, HPIV-3 and RSV subtypes showed distributions that differed from those seen before the pandemic. These findings may be consistent with changes in population immunity and contact patterns, although the present study was not designed to determine the underlying mechanisms directly. Accordingly, monitoring and prevention strategies may need to remain responsive to real-time epidemiologic signals rather than relying solely on fixed seasonal expectations. Future studies should further evaluate these patterns through multi-center validation, molecular epidemiology, and linkage with clinical severity indicators.

## Figures and Tables

**Table 1:** Demographic Characteristics of Pediatric specimens Tested for Respiratory Viruses (2007–2023).

Characteristic	Category	*n*	%
Total		15,657	100
Age Cohort	<1 year old	4556	29.10
	1–6 years old	9317	59.51
	7–12 years old	1207	7.71
	13–18 years old	577	3.69
Sex	Male	9032	57.69
	Female	6625	42.31
Age (years old)	Median (IQR)	1	0.37–3.0

IQR: interquartile range.

**Table 2:** Positivity Rates of Respiratory Viruses in Specimens From Pediatric Patients Adjusted for Panel Coverage (2007–2023).

Virus	Coverage	Total Tested (*n*)	Positive (*n*)	Positivity Rate (%)	95% CI (Wilson)
Inf-A	2007–2023	15,657	699	4.46	4.15–4.80
Inf-B	2007–2023	15,657	287	1.83	1.63–2.06
RSV-A	2007–2023	15,657	1742	11.13	10.64–11.63
RSV-B	2007–2023	15,657	1355	8.65	8.22–9.10
HMPV	2007–2023	15,657	834	5.33	4.99–5.69
HPIV-1	2007–2023	15,657	449	2.87	2.62–3.14
HPIV-2	2007–2023	15,657	155	0.99	0.85–1.16
HPIV-3	2007–2023	15,657	972	6.21	5.84–6.60
HRV	2007–2023	15,657	3767	24.06	23.40–24.74
Cov 229E	2007–2023	15,657	257	1.64	1.45–1.85
OC 43	2007–2023	15,657	426	2.72	2.48–2.99
Adeno	2007–2023	15,657	1930	12.33	11.82–12.85
EV	2018–2023	2355	258	10.96	9.76–12.28
NL63	2015–2023	6552	112	1.71	1.42–2.05
HBoV	2015–2023	6552	402	6.14	5.58–6.74

The positivity rate was calculated as the number of specimens testing positive for each virus divided by the total number of specimens tested for that virus (positive/tested). The full study population (*N* = 15,657) was used as the denominator for viruses included in the assay panel throughout 2007–2023, whereas era-specific denominators were applied for viruses with limited panel coverage (EV, 2018–2023; NL63, 2015–2023; HBoV, 2015–2023). Abbreviations: Inf = influenza virus; RSV = respiratory syncytial virus; HMPV = human metapneumovirus; HPIV = human parainfluenza virus; HRV = human rhinovirus; HCoV = human coronavirus; Adeno = adenovirus; HBoV = human bocavirus; EV = enterovirus. For viruses with limited assay panel coverage, era-specific denominators have been applied: NL63 and human bocavirus (HBoV) from 2015 to 2023 (N = 6552), and enterovirus (EV) from 2018 to 2023 (N = 2355). HCoV includes the subtypes OC 43, 229E, and NL63.

**Table 3:** Monthly Positivity Rates (%) by Respiratory Virus.

Month	Inf-A	Inf-B	RSV-A	RSV-B	HMPV	HPIV-1	HPIV-2	HPIV-3	HRV	Cov229E	OC 43	NL63	Adeno	EV	HBoV	*p* Value
January	12.61	2.18	17.14	16.49	1.29	1.13	0.97	0.89	10.27	3.96	5.50	2.95	8.73	3.66	2.56	<0.001
February	15.08	4.76	11.94	9.92	2.83	1.62	0.61	0.81	13.87	4.55	4.96	4.19	8.10	3.40	5.95	
March	6.28	7.07	6.28	5.50	8.20	2.01	0.09	2.27	24.96	1.48	2.71	5.65	10.91	8.13	3.02	
April	4.75	5.84	4.62	4.81	17.78	2.57	0.26	7.64	27.09	1.03	1.86	2.15	11.81	8.97	10.1	
May	1.69	0.93	1.51	1.63	14.62	2.39	0.58	20.5	26.44	0.93	1.51	1.01	15.14	10.55	14.49	
June	0.59	0.25	1.10	0.51	5.51	2.97	1.10	21.19	29.24	0.25	2.29	0.00	14.41	8.67	11.63	
July	0.59	0.10	0.50	0.3	2.97	4.76	1.09	8.82	35.98	0.20	1.29	0.39	14.47	20.83	8.25	
August	0.00	0.10	2.15	0.92	1.74	6.03	1.33	2.96	27.68	0.51	0.61	0.00	14.61	16.74	6.86	
September	0.97	0.00	4.36	3.49	1.26	5.33	2.13	1.36	33.53	0.48	0.68	0.00	14.24	19.23	3.80	
October	1.35	0.08	15.43	10.02	1.27	4.06	2.70	3.02	30.07	1.43	0.56	0.39	13.44	9.66	1.96	
November	1.77	0.00	27.85	18.35	0.65	2.30	1.12	1.65	21.3	1.65	2.77	0.90	10.44	8.91	2.69	
December	8.02	1.02	26.47	21.3	0.86	1.51	0.54	0.43	14.9	2.85	6.24	2.52	11.89	6.62	2.14	

Abbreviations: Inf = influenza; RSV = respiratory syncytial virus; HMPV = human metapneumovirus; HPIV = human parainfluenza virus; HRV = human rhinovirus; Adeno = adenovirus; EV = enterovirus; HBoV = human bocavirus. HCoV includes the subtypes OC 43, 229E, and NL63. Values indicate monthly positivity rates (% positive tests) for each virus. All viruses exhibit statistically significant monthly variation (*p* < 0.001). Monthly positivity rates for EV have been calculated using data from 2018 to 2023. Monthly positivity rates for NL63 and HBoV have been calculated using data from 2015 to 2023, reflecting limitations in assay panel coverage.

**Table 4:** Age-Cohort–Specific Positivity Across Pre-Pandemic, Pandemic, and Post-Pandemic Recovery Periods.

Age Cohort	Pre-Pandemic Period (2007–2019), *n*/*N* (%) [95% CI]	Pandemic Period (2020–2022), *n*/*N* (%) [95% CI]	Post-Pandemic Recovery Period (2023), *n*/*N* (%) [95% CI]	*p* Value (Across Periods)
<1 year old	2777/4302 (64.55) [63.11–65.97]	96/214 (44.86) [38.35–51.56]	23/40 (57.50) [42.20–71.49]	<0.001
1–6 years old	6587/8853 (74.40) [73.48–75.30]	246/358 (68.72) [63.73–73.30]	90/106 (84.91) [76.88–90.49]	0.00231
7–12 years old	517/1129 (45.79) [42.91–48.71]	20/64 (31.25) [21.23–43.39]	9/14 (64.29) [38.76–83.66]	0.0267
13–18 years old	194/519 (37.38) [33.32–41.62]	8/47 (17.02) [8.89–30.14]	5/11 (45.45) [21.27–71.99]	0.011
*p* value (across age cohorts)	<0.001	<0.001	<0.001	

The 2023 denominators are smaller because period-comparative analyses were restricted to the intersection (complete-case) cohort, which included only specimens with consistently retrievable legacy-panel data and key variables during the 2023 panel transition. Accordingly, post-pandemic estimates are presented with 95% confidence intervals and should be interpreted with attention to wider uncertainty, especially in age groups with small denominators.

**Table 5:** Positivity Rates of 13 Respiratory Viruses Across Pre-Pandemic, Pandemic, and Post-Pandemic Recovery Periods, With Post-Pandemic vs. Pre-Pandemic Effect Sizes.

Virus	Pre-Pandemic Period (*n*/*N*, %)	Pandemic Period (*n*/*N*, %)	Post-Pandemic Recovery Period (*n*/*N*, %)	Omnibus *p* Value (Pre–Pandemic–Pandemic–Post)	Post-Pandemic vs. Pre-Pandemic OR (95% CI)	Post-Pandemic vs. Pre-Pandemic *p* Value
HPIV-3	877/14,803 (5.92)	59/683 (8.64)	36/171 (21.05)	<0.001	4.23 (2.91–6.15)	<0.001
EV	141/1501 (9.39)	80/683 (11.71)	37/171 (21.64)	<0.001	2.66 (1.78–3.99)	<0.001
OC 43	407/14,803 (2.75)	10/683 (1.46)	9/171 (5.26)	0.014	1.97 (1.00–3.87)	0.057
HMPV	813/14,803 (5.49)	10/683 (1.46)	11/171 (6.43)	<0.001	1.18 (0.64–2.19)	0.610
HRV	3565/14,803 (24.08)	158/683 (23.13)	44/171 (25.73)	0.746	1.09 (0.77–1.54)	0.591
Adeno	1881/14,803 (12.71)	31/683 (4.54)	18/171 (10.53)	<0.001	0.81 (0.49–1.32)	0.487
Inf-A	691/14,803 (4.67)	3/683 (0.44)	5/171 (2.92)	<0.001	0.62 (0.25–1.50)	0.361
Inf-B	286/14,803 (1.93)	0/683 (0.00)	1/171 (0.58)	<0.001	0.30 (0.04–2.14)	0.388
HPIV-1	438/14,803 (2.96)	8/683 (1.17)	3/171 (1.75)	0.008	0.59 (0.19–1.84)	0.494
HPIV-2	147/14,803 (0.99)	6/683 (0.88)	2/171 (1.17)	0.861	1.18 (0.29–4.80)	0.689
HBoV	333/5698 (5.84)	56/683 (8.20)	13/171 (7.60)	0.038	1.33 (0.74–2.36)	0.322
Cov 229E	254/14,803 (1.72)	2/683 (0.29)	1/171 (0.58)	0.003	0.34 (0.05–2.42)	0.376
NL63	106/5698 (1.86)	3/683 (0.44)	3/171 (1.75)	0.009	0.94 (0.30–3.00)	1.000

Period comparisons were conducted using the same intersection (complete-case) cohort across pre-pandemic, pandemic, and post-pandemic recovery periods. Because the post-pandemic recovery denominator was substantially reduced during the 2023 panel transition (N = 171), point estimates may be unstable for less frequent viruses; therefore, interpretation should prioritize effect direction and 95% confidence intervals. Abbreviations: Inf = Influenza; HPIV = human parainfluenza virus; HMPV = human metapneumovirus; HRV = human rhinovirus; Adeno = adenovirus; HBoV = human bocavirus; HCoV = human coronavirus; CI = confidence interval; EV = enterovirus. HCoV includes the subtypes OC 43, 229E, and NL63.

**Table 6:** Period-Specific Respiratory Syncytial Virus (RSV)-A and RSV-B Positivity Rates and Odds Ratios (ORs).

Period	RSV-A Positivity % (*n*/*N*) [95% CI]	RSV-B Positivity % (*n*/*N*) [95% CI]	OR (RSV-B Compared with RSV-A)	95% CI	*p* Value
Pre-Pandemic (2007–2019)	11.32 (1675/14,803) [10.81–11.84]	8.65 (1281/14,803) [8.21–9.12]	0.742	0.688–0.802	<0.001
Pandemic (2020–2022)	8.05 (55/683) [6.24–10.34]	8.64 (59/683) [6.76–10.98]	1.08	0.736–1.585	0.6956
Post-Pandemic Recovery (2023)	7.02 (12/171) [4.06–11.86]	8.77 (15/171) [5.39–13.97]	1.274	0.578–2.809	0.5482

The OR represents the odds of RSV-B positivity relative to RSV-A positivity within each period. OR > 1 indicates higher odds of RSV-B positivity, and OR < 1 indicates lower odds of RSV-B positivity.

## Data Availability

The dataset analyzed in this study was derived from laboratory records at Dankook University Hospital and is subject to institutional as well as national ethical guidelines. Owing to confidentiality requirements and data protection regulations, the original raw data cannot be made publicly available. However, de-identified aggregated data may be provided by the corresponding author upon reasonable request and contingent on approval from the Institutional Review Board. All data access inquiries should be directed to the corresponding author.

## Supplementary Materials

The following supporting information can be downloaded at: https://www.mdpi.com/article/10.3390/v18040420/s1, Table S1, STROBE checklist; Table S2, Model-estimated monthly peak timing and seasonal strength of 15 respiratory viruses in the pediatric cohort; Table S3, Pairwise comparisons of positivity rates across study periods within each age cohort; Table S4, False discovery rate–adjusted *p* values for virus-specific comparisons across pre-pandemic, pandemic, and post-pandemic recovery periods; Table S5, Pairwise comparisons of virus-specific positivity rates across study periods.

## Abbreviations

The following abbreviations are used in this manuscript:

AIC	Akaike Information Criterion
95% CI	95% confidence interval
COVID-19	coronavirus disease 19
df	degrees of freedom
DNA	deoxyribonucleic acid
EMR	electronic medical record
EV	enterovirus
HBoV	human bocavirus
HCoV	human coronavirus
HMPV	human metapneumovirus
HPIV	human parainfluenza virus
HRV	human rhinovirus
IQR	interquartile range
NPI	non-pharmaceutical intervention
OR	odds ratio
PCR	polymerase chain reaction
RNA	ribonucleic acid
RSV	respiratory syncytial virus
RT	reverse transcription
USA	United States of America
