# Supplementary material for: Changes in Seasonal Patterns of Pediatric Respiratory Viral Infections Before, During, and After the COVID-19 Pandemic: A Seventeen-Year Surveillance Study in the Republic of Korea
Source: Viruses. 2026 Mar 29;18(4):420. doi: 10.3390/v18040420 (PMC13119541; doi:10.3390/v18040420)
Supplement: Supplementary file 1 [file viruses-18-00420-s001.zip › pediatrics_Table_S2.pdf]

Table S2 Model-estimated monthly peak timing and seasonal strength of 15 respiratory viruses in the pediatric cohort

Virus	Peak month (model)	Seasonal strength (Amplitude, logit)	p value
Inf-A	2	1.84	<0.001
Inf-B	3	3.12	<0.001
RSV-A	11	1.84	<0.001
RSV-B	12	1.82	<0.001
HMPV	4	1.49	<0.001
HPIV-1	8	0.67	<0.001
HPIV-2	9	0.94	<0.001
HPIV-3	5	1.62	<0.001
HRV	9	0.55	<0.001
Cov 229E	1	1.26	<0.001
OC 43	1	0.94	<0.001
Adeno	6	0.27	<0.001
EV	8	0.77	<0.001
NL63	2	1.82	<0.001
HBoV	5	0.98	<0.001

This table summarizes the estimated monthly peak timing and seasonal strength for 15 respiratory viruses based on month-aggregated surveillance data. Peak month (model) indicates the month at which the fitted seasonal curve attains its maximum. Seasonal strength (amplitude, logit) represents the magnitude of seasonal fluctuation on the logit scale, with larger values indicating more pronounced seasonality. Model-estimated peak months may not always match the observed monthly maxima reported in the main text/Table 3 because the fitted harmonic curve summarizes the overall seasonal pattern and may shift the peak toward a neighboring month or the center of a broad seasonal plateau.

Abbreviations: *Inf = influenza virus; RSV = respiratory syncytial virus; HMPV = human metapneumovirus; HPIV = human parainfluenza virus; HRV = human rhinovirus; HCoV = human coronavirus; Adeno = adenovirus; HBoV = human bocavirus; EV = enterovirus.*

For viruses with limited assay panel coverage, era-specific denominators have been applied: HCoV-NL63 and human bocavirus (HBoV) from 2015 to 2023 (N = 6552), and enterovirus (EV) from 2018 to 2023 (N = 2355). HCoV includes the subtypes OC43, 229E, and NL63.
