# Supplementary material for: Changes in Seasonal Patterns of Pediatric Respiratory Viral Infections Before, During, and After the COVID-19 Pandemic: A Seventeen-Year Surveillance Study in the Republic of Korea
Source: Viruses. 2026 Mar 29;18(4):420. doi: 10.3390/v18040420 (PMC13119541; doi:10.3390/v18040420)
Supplement: Supplementary file 1 [file viruses-18-00420-s001.zip › pediatrics_Table_S5.pdf]

Table S5 Pairwise comparisons of virus-specific positivity rates across study periods

Virus	Comparison	OR (95% CI)	p value	Holm-adjusted p value
HPIV-3	Pre-pandemic vs Pandemic	0.67 (0.50–0.89)	0.005	0.005
HPIV-3	Pre-pandemic vs Post-pandemic	0.24 (0.16–0.35)	<0.001	<0.001
HPIV-3	Pandemic vs Post-pandemic	0.36 (0.22–0.58)	<0.001	<0.001
EV	Pre-pandemic vs Pandemic	0.78 (0.58–1.06)	0.108	0.108
EV	Pre-pandemic vs Post-pandemic	0.38 (0.25–0.58)	<0.001	<0.001
EV	Pandemic vs Post-pandemic	0.48 (0.31–0.76)	0.001	0.002
OC 43	Pre-pandemic vs Pandemic	1.90 (1.02–4.02)	0.040	0.079
OC 43	Pre-pandemic vs Post-pandemic	0.51 (0.26–1.14)	0.057	0.079
OC 43	Pandemic vs Post-pandemic	0.27 (0.10–0.76)	0.006	0.018
HMPV	Pre-pandemic vs Pandemic	3.91 (2.10–8.23)	<0.001	<0.001
HMPV	Pre-pandemic vs Post-pandemic	0.85 (0.46–1.73)	0.610	0.610
HMPV	Pandemic vs Post-pandemic	0.22 (0.08–0.57)	<0.001	0.002
HRV	Pre-pandemic vs Pandemic	1.05 (0.88–1.27)	0.614	1.000
HRV	Pre-pandemic vs Post-pandemic	0.92 (0.64–1.32)	0.591	1.000
HRV	Pandemic vs Post-pandemic	0.87 (0.58–1.31)	0.482	1.000
Adeno	Pre-pandemic vs Pandemic	3.06 (2.13–4.56)	<0.001	<0.001
Adeno	Pre-pandemic vs Post-pandemic	1.24 (0.75–2.15)	0.487	0.487
Adeno	Pandemic vs Post-pandemic	0.40 (0.21–0.79)	0.005	0.010
Inf-A	Pre-pandemic vs Pandemic	11.10 (3.76–54.06)	<0.001	<0.001
Inf-A	Pre-pandemic vs Post-pandemic	1.63 (0.68–5.09)	0.361	0.361
Inf-A	Pandemic vs Post-pandemic	0.15 (0.02–0.76)	0.010	0.020
Inf-B	Pre-pandemic vs Pandemic	Inf (3.61–Inf)	<0.001	<0.001
Inf-B	Pre-pandemic vs Post-pandemic	3.35 (0.59–133.46)	0.388	0.400
Inf-B	Pandemic vs Post-pandemic	0.00 (0.00–9.76)	0.200	0.400
HPIV-1	Pre-pandemic vs Pandemic	2.57 (1.29–6.02)	0.005	0.014
HPIV-1	Pre-pandemic vs Post-pandemic	1.71 (0.57–8.39)	0.494	0.935
HPIV-1	Pandemic vs Post-pandemic	0.66 (0.16–3.93)	0.468	0.935
HPIV-2	Pre-pandemic vs Pandemic	1.13 (0.51–3.15)	1.000	1.000
HPIV-2	Pre-pandemic vs Post-pandemic	0.85 (0.23–7.12)	0.689	1.000
HPIV-2	Pandemic vs Post-pandemic	0.75 (0.13–7.65)	0.664	1.000
HBoV	Pre-pandemic vs Pandemic	0.69 (0.52–0.95)	0.018	0.053
HBoV	Pre-pandemic vs Post-pandemic	0.75 (0.42–1.46)	0.322	0.643
HBoV	Pandemic vs Post-pandemic	1.09 (0.57–2.22)	0.876	0.876
Cov 229E	Pre-pandemic vs Pandemic	5.94 (1.62–49.44)	0.002	0.005
Cov 229E	Pre-pandemic vs Post-pandemic	2.97 (0.52–118.32)	0.376	0.752
Cov 229E	Pandemic vs Post-pandemic	0.50 (0.03–29.63)	0.489	0.752
NL63	Pre-pandemic vs Pandemic	4.30 (1.42–21.22)	0.004	0.013
NL63	Pre-pandemic vs Post-pandemic	1.06 (0.35–5.28)	1.000	1.000

Virus	Comparison	OR (95% CI)	p value	Holm-adjusted p value
NL63	Pandemic vs Post-pandemic	0.25 (0.03–1.87)	0.098	0.197

Infinite or zero odds ratios occurred in comparisons with zero counts in one cell; exact confidence intervals were estimated using Fisher's exact method.
