# Supplementary material for: Changes in Seasonal Patterns of Pediatric Respiratory Viral Infections Before, During, and After the COVID-19 Pandemic: A Seventeen-Year Surveillance Study in the Republic of Korea
Source: Viruses. 2026 Mar 29;18(4):420. doi: 10.3390/v18040420 (PMC13119541; doi:10.3390/v18040420)
Supplement: Supplementary file 1 [file viruses-18-00420-s001.zip › pediatric_Table_S3.pdf]

Table S3 Pairwise comparisons of positivity rates across study periods within each age cohort

Age cohort	Comparison	OR (95% CI)	p value	Holm-adjusted p value
<1 year old	Pre-pandemic vs Pandemic	2.24 (1.68–2.98)	<0.001	<0.001
<1 year old	Pre-pandemic vs Post-pandemic	1.35 (0.67–2.64)	0.407	0.407
<1 year old	Pandemic vs Post-pandemic	0.60 (0.28–1.25)	0.168	0.336
1–6 years old	Pre-pandemic vs Pandemic	1.32 (1.04–1.67)	0.019	0.026
1–6 years old	Pre-pandemic vs Post-pandemic	0.52 (0.28–0.89)	0.013	0.026
1–6 years old	Pandemic vs Post-pandemic	0.39 (0.20–0.71)	<0.001	0.002
7–12 years old	Pre-pandemic vs Pandemic	1.86 (1.06–3.37)	0.028	0.083
7–12 years old	Pre-pandemic vs Post-pandemic	0.47 (0.12–1.57)	0.187	0.187
7–12 years old	Pandemic vs Post-pandemic	0.26 (0.06–0.98)	0.032	0.083
13–18 years old	Pre-pandemic vs Pandemic	2.91 (1.30–7.35)	0.006	0.019
13–18 years old	Pre-pandemic vs Post-pandemic	0.72 (0.18–3.01)	0.754	0.754
13–18 years old	Pandemic vs Post-pandemic	0.25 (0.05–1.32)	0.101	0.202
