# Supplementary material for: Changes in Seasonal Patterns of Pediatric Respiratory Viral Infections Before, During, and After the COVID-19 Pandemic: A Seventeen-Year Surveillance Study in the Republic of Korea
Source: Viruses. 2026 Mar 29;18(4):420. doi: 10.3390/v18040420 (PMC13119541; doi:10.3390/v18040420)
Supplement: Supplementary file 1 [file viruses-18-00420-s001.zip › pediatric_Table_S4.pdf]

**Table S4** False discovery rate–adjusted p values for virus-specific comparisons across pre-pandemic, pandemic, and post-pandemic recovery periods

| Virus | FDR-adjusted omnibus p value | FDR-adjusted post-pandemic vs pre-pandemic p value |
|---|---|---|
| HPIV-3 | 0.002 | 0.007 |
| EV | 0.002 | 0.007 |
| OC 43 | 0.018 | 0.247 |
| HMPV | 0.002 | 0.721 |
| HRV | 0.808 | 0.721 |
| Adeno | 0.002 | 0.714 |
| Inf-A | 0.002 | 0.714 |
| Inf-B | 0.002 | 0.714 |
| HPIV-1 | 0.013 | 0.714 |
| HPIV-2 | 0.861 | 0.746 |
| HBoV | 0.045 | 0.714 |
| Cov 229E | 0.006 | 0.714 |
| NL63 | 0.013 | 1.000 |
